# *Cacopsylla fuscicella* Sp. Nov. (Hemiptera, Psyllidae), a New Loquat Pest in China †

**DOI:** 10.3390/insects14050414

**Published:** 2023-04-26

**Authors:** Yisheng Zhao, Xinyu Luo, Yingqi Liu, Anxian Shi, Wanzhi Cai, Fan Song

**Affiliations:** 1 Department of Entomology MOA Key Lab of Pest Monitoring and Green Management, College of Plant Protection, China Agricultural University, Beijing 100193, China; zhaoyisheng@cau.edu.cn (Y.Z.); ltquail@126.com (X.L.); yingqiliu0720@163.com (Y.L.); caiwz@cau.edu.cn (W.C.); 2 Zhaotong Green Food Development Center, Zhaotong, Yunnan 657000, China; ztanxian@163.com

**Keywords:** integrative taxonomy, jumping plant lice, *Eriobotrya japonica*, new species, mitochondrial genome

## Abstract

**Simple Summary:**

*Cacopsylla*, the most species-rich genus of Psyllidae, has around 500 known species worldwide. These insects are known for being host-specific, and some of them are regarded as important pests. Here, we described a new species, *Cacopsylla fuscicella* sp. nov., which develops on loquats. In addition, we sequenced its mitochondrial genome and used these data to construct a phylogenetic tree of Psyllidae, while also calculating the interspecific genetic distances. Using integrative taxonomy methods, we confirmed that the new species, *Cacopsylla fuscicella* sp. nov., should be grouped within genus *Cacopsylla*.

**Abstract:**

Psylloidea, commonly known as jumping plant-lice, have a phloem-sucking habit and strong specificity to their host plants. Within the Psyllidae family, the genus *Cacopsylla* Ossiannilsson, 1970 stands out as being the most diverse, with three species feeding on the genus *Eriobotrya* Lindl. In this study, a new psyllid species, *Cacopsylla fuscicella* sp. nov., was described from China. It is a pest of *Eriobotrya japonica* (Thunb.) Lindl. and has been cultivated as a commercial fruit tree for years. Illustrations of habitus, morphological structures, and its damage to loquat were also provided. The complete mitochondrial genome of *C. fuscicella* sp. nov. was sequenced and annotated. The phylogenetic tree inferred from the maximum likelihood analyses confirmed *C. fuscicella* sp. nov. as a member of the genus *Cacopsylla*. Genetic distances were generated to assess comparative genetic distances within Psyllinae.

## 1. Introduction

The Psylloidea, commonly known as jumping plant lice, are a group of phytophagous insects with a phloem-sucking habit, around 1–6 mm in size. Some species are important pests, damaging crops and trees and even vectoring plant diseases [1]. Generally, psyllids are host-specific species. The range of host plants for a certain psyllid species is usually one genus or several genera belonging to the same family of plant. Currently, *Cacopsylla* Ossiannilsson, 1970 is the most diverse genus in the family Psyllidae, with nearly 500 known species distributed worldwide [2]. Of these, around 400 species have been recorded to occur in China [3]. This genus has been considered polyphyletic by multiple authors, with different species feeding on distinct plant groups, putatively forming many species groups. Consequently, numerous species remain disputed or ungrouped [4,5]. A considerable number of *Cacopsylla* species are known to feed on various Rosaceae plants. These species have historically been assigned to different subgenera [2], but these subgenera are likely not monophyletic and need further clarification. For example, different members feeding on *Pyrus* spp. from western and eastern Palaearctic regions have been considered to be distinctly related. This implies at least two independent origins for these species, which may have evolved as a result of separately occurring host shifting events [6,7]. These thus leave the pattern of evolution and taxonomy of *Cacopsylla* problematic and intriguing. 

Molecular methods have been used to help elucidate problems of taxonomy and evolutionary patterns in insects. Integrative taxonomy is widely regarded as a promising method for resolving taxonomic issues. For example, Cho et al. [8] analyzed DNA barcodes of 11 pear psyllid species to confirm synonymies and correct misidentifications. Mitochondrial genes are commonly used in phylogenetic analyses. Percy et al. [4] used more than 75 complete mitochondrial genomes of Psyllidae taxa in their phylogenetic study, with eight species being annotated and uploaded to GenBank. Similarly, 13 protein coding genes (PCGs) of two and three species of *Cacopsylla* were used to build phylogenetic trees of Psyllidae and Psylloidea, respectively [9,10]. Currently, 12 annotated mitochondrial genomes (mitogenomes) of Psyllidae species are available in GenBank [4,9,10,11,12,13]. 

The *Cacopsylla* species, which are associated with Rosaceae, also include several significant agricultural pests. The Chinese pear psyllid, *Cacopsylla chinensis* (Yang and Li, 1981), for example, has long been a major pest in pear orchards in China [14]; while the West Palearctic species, *Cacopsylla pyricola* (Förster, 1848), is an important pear pest in Europe and also known as the vector of the fire blight [15,16]. Similar concerns are continuously arising with the discovery of more *Cacopsylla* species associated with this fruit-rich family; some species have been treated as local endemic pests [7,17]. 

A rather small group of *Cacopsylla* including three species dealt with in this research feeds on *Eriobotrya* Lindl., a genus of the angiosperm family Rosaceae. The three species are *Cacopsylla eriobotryacola* (Yang, 1984), *Cacopsylla eriobotryae* (Yang, 1984), and *Cacopsylla biwa* (Inoue, 2013). *C. eriobotryacola* and *C. eriobotryae* are associated with the species *Eriobotrya deflexa* (Hemsl.) Nakai; and the other one, *C. biwa*, is the pest of *E. japonica* [18,19]. *Eriobotrya japonica* (Thunb.) Lindl., also known as loquat or biwa, is cultivated for its fruit and has become the most important commercial fruit tree among this genus. It is being increasingly cultivated in Asia, especially in China, where it even became the major income source for the local fruit growers. The new species described here was found in loquat orchards in Zhaotong City, Yunnan Province, where they occur abundantly and cause considerable damage.

In the present study, we describe a new *Cacopsylla* species feeding on loquats, providing characters of both adults and fifth-instar immatures. We also sequenced its mitochondrial genome and compare genetic distances within Psyllinae. A phylogenetic tree using 13 PCGs and 2 rRNA genes of Psyllidae was constructed to discuss the systematic position of *Cacopsylla fuscicella* sp. nov. relative to other species of *Cacopsylla* and Psyllidae sensu stricto. 

## 2. Materials and Methods

### 2.1. Morphological Study

Specimens were boiled in 10% potassium hydroxide (KOH) (Sinopharm Chemical Reagent Co., Ltd., Beijing, China) solution for 10 min, naturally cooled down, washed in distilled water, and mounted on a slide in glycerin (Sinopharm Chemical Reagent Co., Ltd., Beijing, China) for further examination. Measurements were taken from slide-mounted specimens. For adults: BL = total body length measured from anterior margin of vertex to tip of folded fore wing, HW = head width, AL = antennal length, TW = mesoscutum width, WL = fore wing length, and TL = metatibial length. For fifth instar immatures: BL = total body length, HW = head width, AL = antennal length, and FL = fore wing pad length, measured as the distance between transverse tangents of anterior angle and posterior margin. Photos were taken with an Olympus BX41 microscope (Tokyo, Japan) attached to a Cannon 7D camera (Tokyo, Japan). Helicon Focus version 5.3 (Helicon Soft Ltd., Kharkiv, Ukraine) was used for image stacking. The unguitractor of immatures was drawn using Illustrator 2020 (Adobe Systems Inc., San Jose, CA, USA). Line drawings of antenna, male terminalia, paramere, and circumanal ring pore field were undertaken with an Olympus BX41 microscope. Photoshop 2020 (Adobe Systems Inc., USA) was used to edit photos including adjusting background color and cropping without modifying any characters of specimens. All specimens are preserved in the Entomological Museum of China Agricultural University (CAU), Beijing, China. The terminology mainly follows those of Vondráček [20], Ouvrard et al. [21], and Yang et al. [22] for adults and that of White and Hodkinson [23] for immatures. The plant names follow POWO [24].

### 2.2. Sampling and DNA Extraction

Voucher specimens of *Cacopsylla fuscicella* sp. nov. were collected on loquat, *Eriobotrya japonica*, in Zhaotong City, Yunnan Province, China. Most specimens were stored in a refrigerator at −20 °C in 100% ethanol. After being identified based on morphological characters, their genomic DNA was extracted using Dneasy Blood and Tissue Kit (Qiagen, Beijing, China) following the manufacturer’s protocol. Some adult specimens were dry mounted. All specimens are preserved in the Entomological Museum of China Agricultural University (CAU), Beijing, China. 

### 2.3. Mitogenome Sequencing, Assembly, and Annotation

The *COX1* fragment (~610 bp) was amplified by polymerase chain reaction (PCR) with newly designed primers UCOIF (5′-TTTCHACNAACCATAAGGAYATTGG-3′) and UCOIR (5′-TANACTTCTGGGTGTCCAAAAAATCA-3′). Short PCR amplifications were carried out using Qiagen Taq DNA polymerase (Qiagen, Beijing, China) with the following cycling conditions: 5 min at 94 °C, followed by 35 cycles of 50 s at 94 °C, 50 sec at 48–55 °C, and 1–2 min at 72 °C. The final elongation step was continued for 10 min at 72 °C. The PCR products were analyzed by 1.0% agarose gel electrophoresis and then sequenced by Sanger sequencing at Sangon Biotech (Beijing, China).

Libraries setting and sequencing were made by the Beijing Genomics institution (Beijing, China). The BGISEQ-500 library was prepared using genomic DNA with an average insert size of 200–400 bp, and pair-end sequencing with 150 bp in length was performed using the BGISEQ-500 sequencer with the processed libraries. For each library, 3 Gb of clean data were obtained after removing reads containing adaptor contamination poly-Ns (>15 bp Ns) or >75 bp bases with quality score ≤ 3. Clean reads were used in de novo assembly by using IDBA-UD [25], with minimum and maximum k-values of 41 and 141 bp, respectively. To identify the corresponding mitogenome assemblies, the assembled contigs were searched with *COX1* sequence using BLAST with at least 98% similarity. To investigate the accuracy of the assembly, clear reads were mapped onto the obtained mitogenome sequences using Geneious prime 2022.2.2 (http://www.geneious.com/ accessed on 24 April 2022). Offering four species of *Cacopsylla* as reference, the mitochondrial genome was extracted and annotated using MitoZ [26]. The annotation accuracy of 13 protein coding genes and 2 rRNA genes were examined by Geneious prime. The online tRNAscan-SE service (http://lowelab.ucsc.edu/tRNAscan-SE/ accessed on 24 April 2022) was used to confirm the locations of the tRNA genes. The complete mitogenome has been uploaded to GenBank (Table 1). 

### 2.4. Phylogenetic Analysis 

Combining the newly sequenced mitogenome and sequences from GenBank, a total of 12 species from the family Psyllidae were included in our phylogenetic analysis. Two species, *Trioza urticae* from Triozidae and *Calophya schini* from Calophyidae, were selected as outgroups (Table 1). 

The two rRNA genes of each species were separately aligned using the L-INS-I strategy in the MAFFT algorithm [27] and trimmed using trimAl v1.2 [28]. The 13 PCGs of each species were translated into amino acids and then aligned using the MAFFT algorithm individually. The sequences of amino acids were trimmed and back translated based on the original DNA sequences in trimAl v1.2. Thirteen PCGs and two rRNA genes were concatenated using FASconCAT-G v 1.04 [29], while the partition file was obtained. Finally, the dataset of PCGRNA matrix with 12,597 nucleotides was conducted. 

Phylogenetic trees constructed using the Maximum Likelihood (ML) method were implemented in the IQ-TREE web server with a dataset partitioned by genes [30]. The robustness of the tree topology was further assessed by SH-aLRT, and 1000 ultrafast bootstrap replicates were implemented. The models were determined as follows: TIM+F+I+G4 (for *ATP6* and *COX2*), HKY+F+G4 (for *ATP8*), TIM2+F+I+G4 (for *COX1* and *COX3*), TPM3u+F+I+G4 (for *srRNA*), TVM+F+I+G4 (for *ND2*, *ND4*, *ND4L*, *ND5* and *lrRNA*), GTR+F+I+G4 (for *CYTB* and *ND1*), HKY+F+I+G4 (for *ND3*), and TPM3+F+G4 (for *ND6*).

### 2.5. Genetic Distance Analysis

We downloaded *COX1* sequences of 30 *Cacopsylla* species, 4 *Psylla* species, 2 *Arytainilla* species, and 1 *Cyamophila* species from GenBank together with the *COX1* sequence of the new species, *Cacopsylla fuscicella* sp. nov., to calculate the genetic distances within Psyllinae (Table S2). The analysis was conducted in MEGA7 [31]. Sequences alignment was performed and positions containing gaps and missing data were eliminated. There was a total of 435 positions in the final dataset. Pairwise distances were calculated based on the p-distance model. 

## 3. Results

### 3.1. Taxonomy

Suborder: Sternorrhyncha

Superfamily: Psylloidea

Family: Psyllidae Latreille, 1807

Genus: *Cacopsylla* Ossiannilsson, 1970

*Cacopsylla fuscicella* sp. nov.

#### 3.1.1. Diagnosis

*Cacopsylla fuscicella* sp. nov. can be identified by the following combination of characters: genal processes short, about half of median suture of vertex in length; moderately divergent apically, with apices nearly truncate; a width of head about twice as long as the length, the inner-anterior angle of the genae convex.

#### 3.1.2. Description

**Figure 1:**
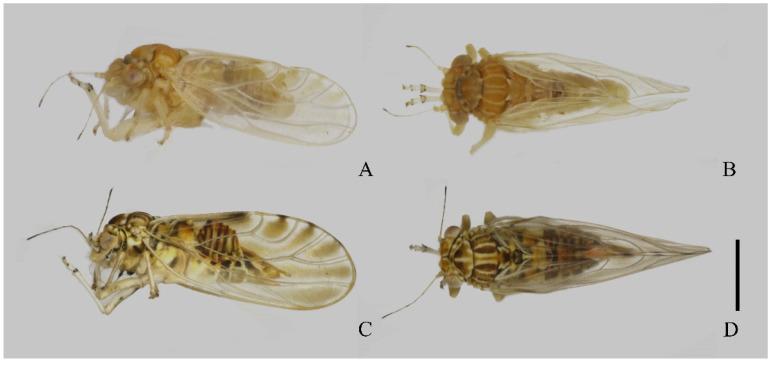
Dry-mounted specimens of *Cacopsylla fuscicella* sp. nov. (**A**). Lateral view of summer form (male). (**B**). Dorsal view of summer form (male) (**C**). Lateral view of winter form (female) (**D**). Dorsal view of winter form (female). Scale bar = 1 mm.

Adult. Coloration. Summer form (Figure 1A,B): Body orange in the overall view. Vertex mostly orange with white anterior angles. Discal foveae light brown in color. Genal process yellow and lighter than body color. Compound eyes yellow. Lateral ocelli and medial ocellus orange. Occiput black, the postocular sclerite white with black margins. Antennae yellow, with segment ten entirely black. Thoracic dorsum orange in ground with white narrow stripes; pronotum four white markings; one longitudinal white stripe present through the middle of the mesopraescutum. Legs yellow, irregular markings on femora darker. Fore wing membrane hyaline, apices of cells m_1_, m_2_ and cu_1_ with ocherous clouding. Veins near yellow, slightly darker than membrane. Hind wing membrane translucent, more or less brownish along veins; veins light brown to brown. Abdominal terga dark brown with margins of segments III–VI white and segment VI yellowish. Male terminalia orange. Female proctiger ochreous, subgenital plate white with irregular dark brown spots. 

Winter form (Figure 1C,D): overall color ochreous, darker than summer form. Fore wing with apical halves of cells r_1_, r_2_, m_1_, m_2_, cu_1_, and cu_2_, dark brown. Posterior margin of each abdominal tergite dark brown.

**Figure 2:**
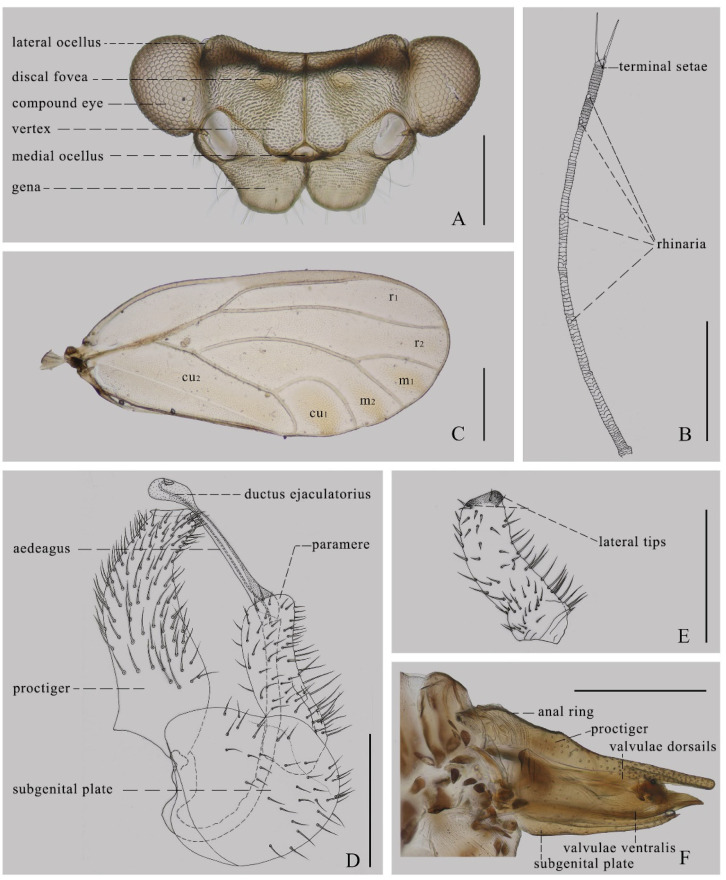
(**A**) Head, frontal aspect; (**B**) antenna; (**C**) forewing of summer form; (**D**) male terminalia, lateral aspect; (**E**) paramere, inner surface; (**F**) female terminalia, lateral aspect. Scale bars = 0.2 mm for (**A**,**D**,**E**); 0.4 mm for (**B**,**F**); 0.5 mm for (**C**,**E**).

Structures. Head inclined from the longitudinal body axis by 80–90°, slightly wider than mesoscutum (Figure 1). Lateral parts of vertex short longitudinally; antero-inner angles of vertex produced and rounded; areas around lateral ocelli strongly convex, appearing distinctly contrasted from areas around discal foveae; vertex and gena clearly separated by a suture (Figure 2A). Discal foveae relatively shallow (Figure 2A). Surface of the vertex finely sculptured with scaly microstructuring and microscopic setae (Figure 2A). Genal processes short, at about half of the median suture of vertex in length; moderately divergent apically, with apices being nearly truncated (Figure 2A). Antennae relatively slender, longer than head width, with a single subapical rhinarium on each of segments 4, 6, 8, and 9; the distally situated terminal seta only slightly shorter than the proximally situated terminal seta (Figure 2B). Mesopraescutum moderately protruding forward to force pronotum to arch (Figure 1B,D). Genual spine of metatibia relatively small, apical spurs of metatibia arranged in 1+1+2+1 (Figure 1B). Fore wing oblong oval, widest in apical 1/3; surface spinules covering most of the fore wing membrane, leaving narrow spinule-free bands along veins (Figure 2C). 

Male terminalia. Whole proctiger gently arched in lateral aspect, covered with near-evenly spaced setae (Figure 2D). Paramere stout, gently curved; apex of paramere axe-like, curved inwards, with two lateral tips better sclerotized (Figure 2D,E). Inner surface of paramere with many erect short setae (Figure 2E). Distal segment of aedeagus relatively long, slender, and nearly straight; apical dilatation small, near oval, and not hooked; sclerotized end tube of ductus ejaculatorius projected backwards and gently curved upwards (Figure 2D). Subgenital plate subtriangular in profile (Figure 2D). 

Female terminalia. The shape is relatively long and simple. The anal ring has a length of about 2/5 of the total length of the proctiger; the dorsum of the proctiger is slightly sinuous in profile. The subgenital plate is relatively long and narrow in profile. Valvulae dorsails and ventralis gently curve upwards and lack teeth (Figure 2F).

Fifth instar immature. Coloration. Generally yellow to orange in color. Membranous parts orange. All sclerites dark brown. Compound eyes red. 

**Figure 3:**
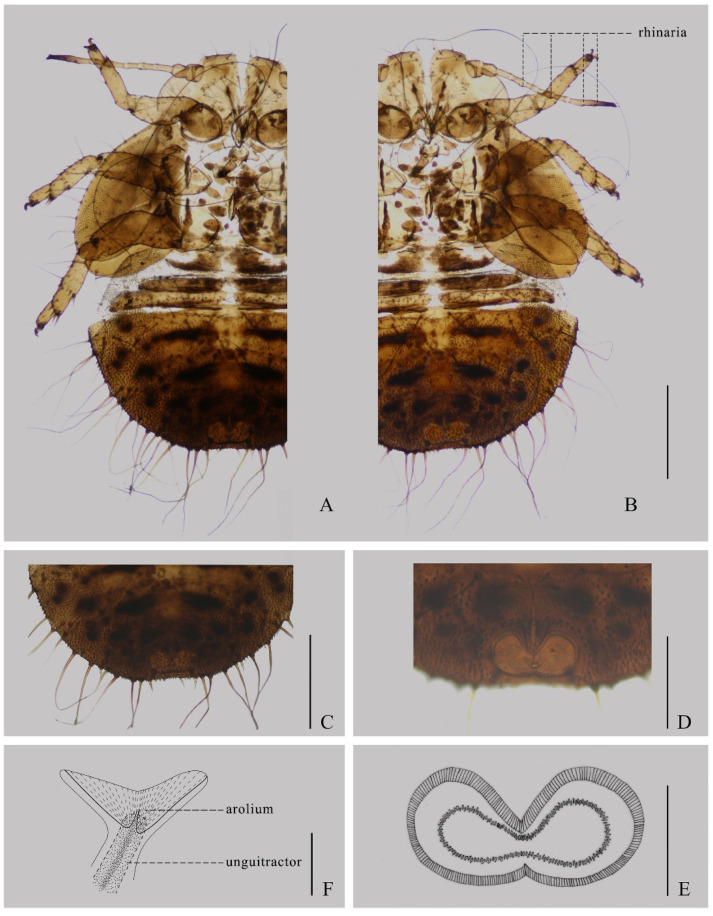
(**A**) fifth instar immature, ventral aspect; (**B**) fifth instar immature, dorsal aspect; (**C**) abdominal sectaseta; (**D**,**E**) circumanal ring, with an outer and inner ring of pores. F. tarsal arolium; Scale bars = 0.5 mm for (**A**–**C**); 0.4 mm for (**D**); 0.2 mm for (**E**); 0.025 mm for (**F**).

Structures. Dorsum completely covered with minute to long simple setae, including ocular and postocular setae (Figure 3A,B). All bases of setae strongly sclerotized. Antenna 8-segmented, with one rhinarium on each apex of segments 4 and 6, and two rhinaria on segment 8 (Figure 3B). Fore wing pad oval in chape, with about ten long setae on the outer margin, a knife-shaped hind wing pad with two long setae on the apex (Figure 3A,B). Abdominal ventrum with long or short conical setae near the margin; other parts with long or short simple setae (Figure 3C). Abdominal margin with rather long 7+7 setae and 3+3 sectasetae (Figure 3C). Circum anal pore field ventral, composed of a complete outer ring of a single row of slit-shaped pores and a complete inner ring of a jagged single row of oval pores (Figure 3D,E). Dorsum of the fore tibial-tarsus with one long seta; dorsum of mid and hind tibial-tarsus with two long setae (Figure 3A,B). Tarsal arolium broadly fishtail shaped with expanded areas rather coarse; pedicel relatively short, gradually narrows basally, and with a well-developed unguitractor (Figure 3F).

#### 3.1.3. Measurements

Measurements are in mm. Adult: ♂ (*n* = 5): BL 3.24–3.51, AL 1.20–1.26, HW 0.89–0.93, TW 0.91–0.96, WL 2.63–2.82, TL 0.62–0.69. ♀ (*n* = 4): BL 3.66–3.87, AL 1.22–1.26, HW 0.87–0.90, TW 0.86–0.89, WL 3.06–3.24, TL 0.64–0.70. Immature: (*n* = 3). BL 1.94–2.18, HW 0.79–0.82, AL 0.83–0.86, FL 0.79–0.85. 

#### 3.1.4. Material Examined

Holotype: male, dry mounted, Zhaotong City, Yunnan Province, China, 30 October 2022, Shi Anxian (CAU). Paratypes: 2 males, 2 females, 1 immature, dry mounted, same data as the holotype; 2 males, 2 females, 1 immature, dry mounted, Zhaotong City, Yunnan Province, China, 13 May 2022 (CAU). 

#### 3.1.5. Distribution

China: Yunnan Province. Known only from the type locality for now.

#### 3.1.6. Host Plant and Damage

**Figure 4:**
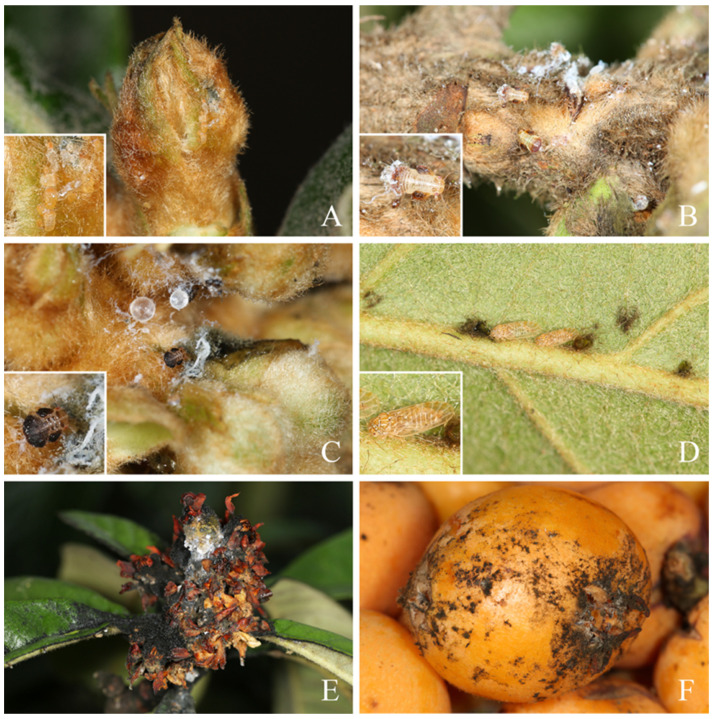
Damage of *Cacopsylla fuscicella* sp. nov. to *Eriobotrya japonica*. (**A**) Nymphs aggregating on base of young leaves; (**B**) Nymphs aggregating on stem; (**C**) Nymphs aggregating on bud; (**D**) Adults aggregating on the undersurface of leaves; (**E**) Damage to flowers; (**F**) Damage to fruits.

The host plant of this new species *Eriobotrya japonica*, which is commonly known as loquat. This plant is native to China and is grown commercially for its fruit. As one of the insect pests in the loquat industry in China, it caused great economic losses to fruit growers. They always aggregate on the undersurface of leaves, stem, and bud, sucking phloem sap, wilting the young leaves and flowers, and causing fruit russet (Figure 4).

#### 3.1.7. Etymology

This species is named after its ocherous cells on the forewings of the summer form. 

#### 3.1.8. Comments

Before the present study, there have been three psyllid species recorded feeding on the plant genus *Eriobotrya* [18,19]. Two among them, *Cacopsylla eriobotryacola* and *C. eriobotryae*, are the pests of *Eriobotrya deflexa*. They have yellowish forewings without ocherous clouding and long and thin parameres. Their genae processes are long and have the same length as the median suture of the vertex. For *C. eriobotryacola*, the gena is thin and acute apically, which can be clearly distinguished from *C. fuscicella* sp. nov. The other species, *C. biwa*, is more similar to *C. fuscicella* sp. nov., both in morphological characters and host plant. They both feed on loquat and *Eriobotrya japonica* and have a similar color pattern; however, there are still some differences. The head of *C. fuscicella* sp. nov. is wider than that of *C. biwa*. The width of the head is about twice as long as the length of head in *C. fuscicella* sp. nov., but the length–width ratio of the head for *C. biwa* is lower than two. For *C. fuscicella* sp. nov., the inner-anterior angle of the genae is convex, but that of *C. biwa* is flat. The genae of *C. fuscicella* sp. nov. are shorter and nearly truncate apically. The setae on the caudal plate of *C. fuscicella* sp. nov. are conspicuously longer than those of *C. biwa*. In the summer form, the color on the cell of r_2_ of *C. fuscicella* is lighter than that of *C. biwa*. A key to *Cacopsylla* species associated with *Eriobotrya* (Rosaceae) was offered as follows.

Key to adults of *Cacopsylla* species associated with *Eriobotrya* (Rosaceae)
1Genae thin and acute apically*C. eriobotryacola*-Not as above22Genae square, male proctiger only weakly curved proximally*C. eriobotryae*-Genae rounded, male proctiger strongly curved proximally33The length-width ratio of the head less than 2, the inner-anterior angle of the genae flat*C. biwa*-Width of head about twice as long as the length, the inner-anterior angle of the genae convex*C. fuscicella* sp. nov

### 3.2. General Features of Cacopsylla fuscicella sp. nov. Mitogenome

The mitogenome of *Cacopsylla fuscicella* sp. nov. was obtained successfully with a length of 14,832 bp, including 37 coding genes (13 PCGs, 2 rRNA genes, and 22 tRNA genes) and a putative control region (CR) (Table 2, Figure S1). Twenty-three genes were transcribed from the majority strand (J-strand) and the other fourteen genes were transcribed from the minority strand (N-strand). The gene order and orientation were identical with the hypothetical ancestral mitogenome without gene rearrangement. Eight gene overlaps comprising a total of thirty-three bp were identified. In addition to the CR, we also observed 10 non-coding regions ranging from 1 to 29 bp (Table 2). The entire mitogenome was biased towards A and T, with the AT content being 70.9%. The nucleotide skew analysis revealed a positive AT skew (0.075) and a negative CG skew (−0.306) (Table S1). 

The total length of the 13 PCGs is 10,768 bp. The protein coding genes have 4 types of start codons (6 ATGs, 4 ATAs, 2 TTGs and 1 ATC), and most started with ATN, except ND5 and ND4L. Nine PCGs stopped with TAA or TAG, while COX2, COX3, ND4, and ND5 truncated termination codons with a single T (Table 2). All 22 tRNA genes were detected and found to be discontinuously scattered throughout the mitogenome. The size of each tRNA gene ranges from 54 bp to 70 bp. The lrRNA gene was 1,102 bp long and was found between trnL1 and trnV, and the srRNA gene, 774 bp in size, was located between trnV and the control region (Table 2, Figure S1). The control region of *Cacopsylla fuscicella* sp. nov. located between srRNA and trnI was 711 bp in length with the highest AT content (79.1%) in the entire mitogenome (Figure S1). There were no notable repeat elements found in the control region. 

### 3.3. Phylogenetic Analyses and Genetic Distance Analyses

**Figure 5:**
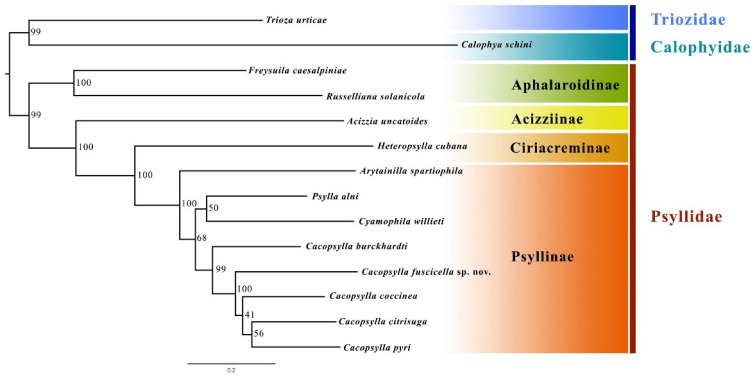
Phylogenetic relationship of Psyllidae inferred via IQ-TREE based on mitochondrial genome sequences. Numbers close to the branching points are bootstrap support values.

The phylogenetic topology of Psyllidae inferred by the ML method is shown in Figure 5. The monophyly of Aphalaroidinae and Psyllinae was recovered with high support values, and Aphalaroidinae was recovered as the sister group to the other three subfamilies. The results further confirm that the new species described in the present study should be placed in the genus *Cacopsylla*. The new species, *Cacopsylla fuscicella* sp. nov., was grouped together with the other four *Cacopsylla* species, and the sister relationship between *C. fuscicella* sp. nov. and the clade (*C*. *coccinea* + (*C*. *citrisuga* + *C*. *pyri*)) was strongly supported (node support value = 100).

The results of the genetic distance analysis (Table S2) showed that the average interspecific genetic distance was 0.171 among the 31 *Cacopsylla* species, 0.161 among the four *Psylla* species, and 0.177 between the 2 *Arytainilla* species. The intergeneric distance within Psyllinae was relatively higher with an average value of 0.194 (*Arytainilla*-*Cacopsylla*: 0.193, *Arytainilla*-*Cyamophila*: 0.189; *Arytainilla*-*Psylla*: 0.219; *Cacopsylla-Cyamophila*: 0.192; *Cacopsylla*-*Psylla*: 0.191; *Cyamophila*-*Psylla*: 0.178). As for the new species, the average genetic distance between *Cacopsylla fuscicella* sp. nov. and the other 30 *Cacopsylla* species was 0.204. However, it is 0.224, 0.221, and 0.216 between *Cacopsylla fuscicella* sp. nov. and *Psylla*, *Arytainilla*, and *Cyamophila*, respectively.

## 4. Discussion

Based on morphological characters, *Cacopsylla fuscicella* sp. nov. can be differentiated from other loquat psyllids by its rounded gena, convex inner anterior angle of genae, and wide head. The degree of DNA barcode also supports the classification of *Cacopsylla fuscicella* sp. nov. as a new species. It is interesting that the divergence between *Cacopsylla fuscicella* sp. nov. and *C. eriobotryae* is not less than divergence between *Cacopsylla fuscicella* sp. nov. and other Rosaceae-feeding species, which suggests that the two species on *Eribotrya* are not particularly closely related. Moreover, the divergence between *Cacopsylla fuscicella* sp. nov. and *C. eriobotyrae* is greater than that between certain two species from different genera, such as *Arytainilla gredi* (Ramirez Gomez, 1956) and *Psylla buxi* (Linnaeus, 1758). This suggests that COI divergence is likely becoming saturated and not a reliable tool for phylogenetic analysis. Therefore, the complete mitochondrial genome is relatively more reliable for confirming systematic placement. Based on the phylogenetic tree we constructed, *Cacopsylla fuscicella* sp. nov. was grouped within the monophyletic *Cacopsylla* clade with high support. The relationship of these four subfamilies is congruent with previous phylogenetic analyses of Psylloidea [5]. 

Psyllids pose a significant threat to crop production and the health of fruit trees, resulting in considerable economic losses. They can deplete plant nutrients, cause tissue damage, and transmit plant pathogens—all of which can have a severe impact on agriculture. The new species, feeding on *Eriobotrya japonica*, was wildly discovered in the loquat orchard in its type locality. This species can cause damage to foliage and flowers in both the immature and adult stages, as well as impede fruit development and cause injury. Therefore, the production and quality of loquat suffered a strong impact, and it causes damage to the industry of loquat. However, there have been insufficient studies conducted on this species, particularly in the area of taxonomy, which hinders efforts to control the pests and conduct further research. Thus, our study may fill the void by providing morphological and molecular data of *Cacopsylla fuscicella* sp. nov. Our work was expected to help investigate the biological characteristics of this species, which in turn would enable us to more accurately monitor the population of the pest. With this information, we could develop more effective control strategies and improve our ability to manage the pest population, ultimately reducing the damage it causes to loquats and other affected areas.

## Figures and Tables

**Table 1:** Taxa used in phylogenetic analysis.

Family	Subfamily	Species	Genbank Accession Number
Triozidae	Triozinae	*Trioza urticae* (Linné, 1758)	NC_038113
Calophyidae	Calophyinae	*Calophya schini* Tuthill, 1959	MF431591
Psyllidae	Aphalaroidinae	*Freysuila caesalpiniae* (Tuthill, 1959)	MG989225
		*Russelliana solanicola* (Tuthill, 1959)	NC_038140
	Acizziinae	*Acizzia uncatoides* (Ferris & Klyver, 1932)	NC_038146
	Ciriacreminae	*Heteropsylla cubana* (Crawford, 1914)	MG989226
	Psyllinae	*Arytainilla spartiophila* (Foerster, 1848)	NC_038133
		*Psylla alni* (Linné, 1758)	NC_038139
		*Cyamophila willieti* (Wu, 1932)	MN364946
		*Cacopsylla burckhardti* (Luo, Li, Ma & Cai, 2012)	OK574466
		*Cacopsylla fuscicella* sp. nov.	OQ390047
		*Cacopsylla coccinea* (Kuwayama, 1908)	NC_027087
		*Cacopsylla citrisuga* (Yang & Li, 1984)	NC_053749
		*Cacopsylla pyri* (Linné, 1758)	NC_038148

**Table 2:** Organization of *Cacopsylla fuscicella* sp. nov. mitochondrial genome.

Gene	Direction	Location	Size (bp)	Anticodon	Codon Start	Codon Stop	Intergenic Nucleotides
*tRNA^Ile^*	F	1–66	66	GAT			
*tRNA^Gln^*	R	71–136	66	TTG			4
*tRNA^Met^*	F	143–208	66	CAT			6
ND2	F	209–1180	972		ATG	TAA	0
*tRNA^Trp^*	F	1203–1267	65	TCA			22
*tRNA^Cys^*	R	1271–1333	63	GCA			3
*tRNA^Tyr^*	R	1334–1395	62	GTA			0
COI	F	1397–2929	1533		ATG	TAA	1
*tRNA^Leu(UUR)^*	F	2929–2996	68	TAA			−1
COII	F	2997–3660	664		ATA	T--	0
*tRNA^Lys^*	F	3661–3730	70	CTT			0
*tRNA^Asp^*	F	3737–3802	66	GTC			6
ATP8	F	3803–3955	153		ATA	TAG	0
ATP6	F	3949–4623	675		ATG	TAA	−7
COIII	F	4623–5400	778		ATG	T--	−1
*tRNA^Gly^*	F	5401–5458	58	TCC			0
ND3	F	5459–5809	351		ATA	TAG	0
*tRNA^Ala^*	F	5808–5868	61	TGC			−2
*tRNA^Arg^*	F	5870–5930	61	TCG			1
*tRNA^Asn^*	F	5931–5997	67	GTT			0
*tRNA^Ser(AGN)^*	F	5998–6051	54	GCT			0
*tRNA^Glu^*	F	6052–6113	62	TTC			0
*tRNA^Phe^*	R	6102–6164	63	GAA			−12
ND5	R	6165–7782	1618		TTG	T--	0
*tRNA^His^*	R	7783–7843	61	GTG			0
ND4	R	7844–9086	1243		ATG	T--	0
*ND4L*	R	9080–9367	288		TTG	TAG	−7
*tRNA^Thr^*	F	9369–9429	61	TGT			1
*tRNA^Pro^*	R	9430–9491	62	TGG			0
*ND6*	F	9494–9979	486		ATC	TAA	2
CytB	F	9979–11,115	1137		ATG	TAG	−1
*tRNA^Ser(UCN)^*	F	11,114–11,175	62	TGA			−2
ND1	R	11,205–12,119	915		ATA	TAA	29
*tRNA^Leu(CUN)^*	R	12,120–12,183	64	TAG			0
lrRNA	R	12,184–13,285	1102				0
*tRNA^Val^*	R	13,286–13,347	62	TAC			0
srRNA	R	13,348–14,121	774				0
Control region		14,122–14,832	711				0

* Negative numbers indicate that adjacent genes overlap.

## Data Availability

The mitochondrial genome of *Cacopsylla fuscicella* sp. nov. has deposited in the GenBank under the accession number OQ390047.

## Supplementary Materials

The following supporting information can be downloaded at: https://www.mdpi.com/article/10.3390/insects14050414/s1, Figure S1. Mitochondrial genome of Cacopsylla fuscicella sp. nov. sequenced in this study. Circular maps were drawn with Geneious prime 2022.2.2 (http://www.geneious.com/ accessed on 4 April 2022). The transcriptional direction is indicated with arrows. Abbreviations: ATP6 and ATP8 for adenosine triphosphate (ATP) synthase subunits 6 and 8; COX1–COX3 for cytochrome oxidase subunits 1–3; CYTB for cytochrome b; ND1–6 and ND4L for nicotinamide adenine dinucleotide hydrogen (NADH) dehydrogenase subunits 1–6 and 4L; lrRNA and srRNA for large and small rRNA subunits; trnX (where X is replaced by one letter amino acid code of the corresponding amino acid), for transfer RNA. Table S1. The nucleotide composition of the Cacopsylla fuscicella sp. nov. mitochondrial genome; Table S2. Genetic distances calculated using p-distance model for COX1 sequences.
