# Supplementary material for: Cacopsylla fuscicella Sp. Nov. (Hemiptera, Psyllidae), a New Loquat Pest in China
Source: Insects. 2023 Apr 26;14(5):414. doi: 10.3390/insects14050414 (PMC10231073; doi:10.3390/insects14050414)
Supplement: Supplementary file 1 [file insects-14-00414-s001.zip › Figure S1.pdf]

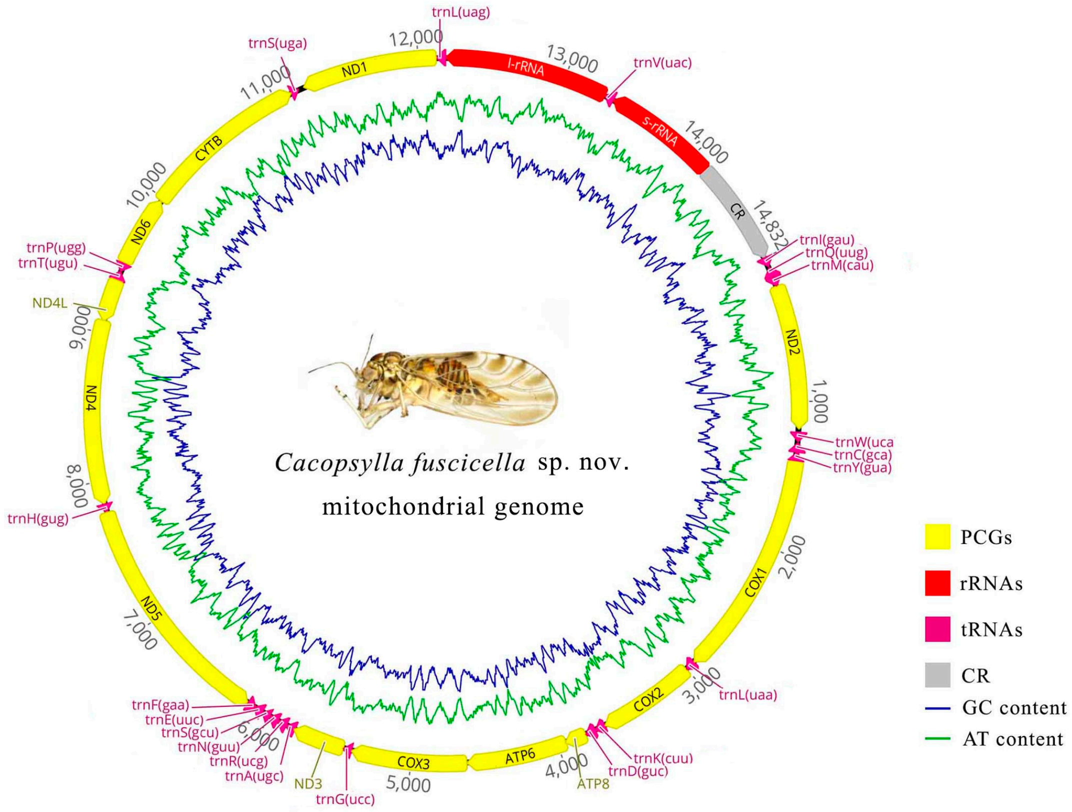

Figure S1. Mitochondrial genome of Cacopsylla fuscicella sp. nov. sequenced in this study. Circular maps were drawn with Geneious prime 2022.2.2 (http://www.geneious.com/ accessed on 4 April 2022). The transcriptional direction is indicated with arrows. Abbreviations: ATP6 and ATP8 for adenosine triphosphate (ATP) synthase subunits 6 and 8; COX1–COX3 for cytochrome oxidase subunits 1–3; CYTB for cytochrome b; ND1–6 and ND4L for nicotinamide adenine dinucleotide hydrogen (NADH) dehydrogenase subunits 1–6 and 4L; lrRNA and srRNA for large and small rRNA subunits; trnX (where X is replaced by one letter amino acid code of the corresponding amino acid), for transfer RNA.
