# Supplementary material for: Cacopsylla fuscicella Sp. Nov. (Hemiptera, Psyllidae), a New Loquat Pest in China
Source: Insects. 2023 Apr 26;14(5):414. doi: 10.3390/insects14050414 (PMC10231073; doi:10.3390/insects14050414)
Supplement: Supplementary file 1 [file insects-14-00414-s001.zip › Table S1 20230330.pdf]

Table S1. The nucleotide composition of the *Cacopsylla fuscicella* sp. nov. mitochondrial genome

	A %	T%	C%	G%	AT content	CG content	AT skew	GC skew
Whole genome	38.1	32.8	19.0	10.1	51.8	29.1	0.075	-0.306
PCGs	29.3	39.7	16.7	14.3	69.0	31.0	-0.151	-0.077
ATP6	33.6	36.1	20.6	9.6	69.7	29.9	-0.036	-0.364
ATP8	32.7	36.1	21.8	9.3	68.8	31.1	-0.049	-0.402
COI	30.5	34.6	21.1	13.8	65.1	34.9	-0.063	-0.209
COII	34.6	31.6	22.0	11.7	66.2	33.7	0.045	-0.306
COIII	31.9	37.0	20.2	10.9	68.9	31.1	-0.074	-0.299
Cytb	30.7	36.9	20.5	11.9	67.6	32.4	-0.092	-0.265
ND1	23.6	46.2	9.9	20.2	69.8	30.1	-0.324	0.342
ND2	39.2	39.2	18.9	6.6	67.6	25.5	-0.052	-0.482
ND3	31.1	37.9	19.9	11.1	69.0	31.0	-0.999	-0.284
ND4	23.9	47.2	9.7	19.2	71.1	28.9	-0.327	0.329
ND4L	24.0	50.7	6.9	18.4	74.7	25.3	-0.357	0.455
ND5	24.5	42.5	11.6	21.4	67.0	33.0	-0.279	0.297
ND6	8.0	37.2	20.8	34.0	71.2	54.8	-0.045	-0.444
16S	35.4	40.1	8.0	16.5	75.5	24.5	-0.062	0.347
12S	35.8	40.4	8.0	15.9	76.2	23.9	-0.060	0.331
CR	39.7	39.4	12.8	8.2	79.1	21.0	0.004	-0.219
